# Promoting latrine construction and use in rural villages practicing open defecation: process evaluation in connection with a randomised controlled trial in Orissa, India

**DOI:** 10.1186/1756-0500-7-486

**Published:** 2014-08-01

**Authors:** Sophie Boisson, Peppin Sosai, Shubajyoti Ray, Parimita Routray, Belen Torondel, Wolf-Peter Schmidt, Bishakha Bhanja, Thomas Clasen

**Affiliations:** 1 Faculty of Infectious and Tropical Diseases, London School of Hygiene & Tropical Medicine, London, United Kingdom; 2 Xavier Institute of Management, Bhubaneswar, India; 3 WaterAid, Bhubaneswar, India; 4 Department of Environmental Health, Rollins School of Public Health, Emory University, Atlanta, GA, USA

**Keywords:** Sanitation, Total Sanitation Campaign, Cluster-randomised trial, Process evaluation

## Abstract

**Background:**

Our group conducted a cluster-randomised trial in 100 villages of Orissa, India to measure the impact of a rural sanitation intervention implemented under the government of India's Total Sanitation Campaign, on diarrhoea and soil-transmitted helminth infections. This paper reports on a process evaluation conducted in the context of the trial.

**Methods:**

Process evaluation data were collected through review of key documentation, quantitative surveys, direct observations, and semi-structured interviews with staff from implementing NGOs and community members. Between March 2011 and March 2012, trained enumerators recorded observations on latrine construction status every 6–8 weeks in the 50 intervention villages and noted activities reported to have taken place based on NGO staff interviews and review of NGO records. A survey among 10% of households in intervention and control villages was conducted to compare levels of awareness of key intervention components. In addition, 10% of village water and sanitation committee (VWSC) members were interviewed to measure their level of involvement in the intervention delivery.

**Results:**

The percentage of households with a latrine (completed or under construction) increased from 8% at baseline to 66% one year after the start of the intervention in March 2012. Almost none of the intervention households recall any form of participatory community-level activities at the start of the programme, although intervention households were generally more aware of the Total Sanitation Campaign (91% versus 49%, p < 0.001), VWSCs (51% versus 9%, p < 0.001), adolescent girls groups (23% versus 8%, p < 0.01), wall paintings (44% versus 7%, p < 0.001) and were more likely to report a household visit on sanitation during the past three months (65% versus 3%, p < 0.001). We found no strong evidence of an association between levels of awareness of or participation in mobilisation activities and levels of latrine coverage in intervention villages.

**Conclusions:**

The levels of coverage achieved and the levels of awareness of the mobilisation process in our intervention villages were lower than planned, but similar to those reported elsewhere in India under the TSC. Our process evaluation highlights important gaps between the TSC guidelines and their implementation on the ground.

**Trial registration:**

Number on clinicaltrial.gov: NCT01214785

## Background

Worldwide, 2.5 billion people or one in four do not have access to improved sanitation. Of these, an estimated 1 billion still practice open defecation [1]. Despite large-scale programmes over the past decades, India represents the largest sanitation challenge. An estimated 626 million people continue to practice open defecation, especially in rural area where only 24% of the county’s rural population have access to improved sanitation facilities [1].

Inadequate sanitation is associated with significant morbidity from diarrhoeal disease, soil-transmitted infection, trachoma, and malnutrition [2]. Systematic reviews have found that sanitation interventions could reduce this disease burden, but the studies reviewed were often of poor quality or measured the effect of sanitation in combination with other water and hygiene interventions [3-5]. In this context, our group undertook a cluster-randomised trial among 100 villages of a coastal district of Orissa to measure the impact of household latrines on diarrhoea, soil-transmitted helminth infections and anthropometrics [6]. Following a baseline survey, 100 villages selected with government cooperation were randomly allocated into two study arms, one to receive the intervention immediately and the other following the end of a 21-month surveillance period.

Implementation of the intervention was led by Wateraid India, a national affiliate of the UK-based NGO widely recognised for its work in water, sanitation and hygiene (wateraid.org) in collaboration with local NGOs. Fieldwork was conducted in the 50 intervention villages over a 14-month period beginning in February 2011. Implementation followed the government of India’s Total Sanitation Campaign (TSC). The TSC, recently expanded and renamed as Nirmal Bharat Abhiyan (NBA), was set up in 1999 to promote toilet construction and use in rural areas. The TSC programme provided subsidies for latrine construction to households who fall below the poverty line (BPL); it also uses community mobilisation and information, education and communication (IEC) activities to create demand and encourage latrine use [7].

Process evaluation can provide some explanations for the level of effectiveness achieved by an intervention [8,9]. Comprehensive process documentation is essential in order to avoid wrongly ascribing the results of an evaluation to an intervention as planned instead of as delivered [10]. This paper reports on the results of a process evaluation of the sanitation intervention delivered in connection with our trial.

## Methods

The design and methods for the trial are described elsewhere in details [6]. The process evaluation component was designed based on the framework developed by Linnan and Steckler [10]. The key objectives of this evaluation were to 1) provide information on the context in which the intervention was implemented 2) document how the intervention was delivered 3) assess exposure to the intervention among the target population, and 4) explore associations between household exposure to community mobilisation activities and construction of latrines.

Process evaluation data were collected through review of key documentation, quantitative surveys, direct observations, and semi-structured interviews with NGOs staff and community members. After an initial review of implementation guidelines and reports on the Total Sanitation Campaign, we met with the implementing NGOs to obtain detailed accounts on what the intervention consisted of at their level of operation. This information was used to develop the data collection tools.

Between March 2011 and March 2012, a team of four trained enumerators and one supervisor visited each of the 50 intervention villages approximately every 6–8 weeks, resulting in six data collection rounds for each village. At each visit, field enumerators conducted the following activities: 1) they interviewed NGO village motivators to obtain information on the campaign activities conducted in the village 2) they reviewed documentation maintained by the village motivators and village water and sanitation committee (VWSC) members such as activity log books, meetings notes, household contribution registers, and construction material stock registers 3) they visited each household to observe and record latrine construction status. Latrine construction status was categorised as ‘completed’ and ‘under construction’. A latrine was classified as ‘completed’ when it met the specification provided by WaterAid. A completed latrine had walls over 1.5 meters, a door, an unbroken and unblocked toilet pan, and a functional pan-pipe-pit connection. Latrine classified as ‘under construction’ were latrines that were left unfinished or latrine that were completed, but subsequently damaged. Between January and March 2013, latrine coverage was assessed in both intervention and control villages.

Between January and June 2012, a survey was conducted among a random sample of 10% of households in each of the 50 control and 50 intervention villages (approximately 400 households in each arm). The male or female head of household or if absent, a household member over sixteen years of age present at the time of visit was asked questions to measure their level of awareness about community mobilisation events undertaken within their village. For each intervention village where a village water and sanitation committee had been formed, we obtained a list of the VWSC members along with basic demographic characteristics. Approximately 10% of VWSC members or two members per village were randomly selected from the list and administered a short questionnaire to assess their involvement in the programme activities such as meetings, attendance to training, and awareness of their role and responsibilities as VWSC members. The sample size for both household and VWSC member surveys was based on logistical considerations. For each village, a list of households and VWSC members was available. A sample was randomly selected from the list using the random generator function in Stata 13.

Questionnaires and interview guides were developed in English, translated into Oriya and back-translated into English to ensure accuracy of translation. Quantitative data were analysed in Stata 13 (Stata corp, College Station, TX). We compared levels of awareness of key mobilisation activities between control and intervention villages. We first calculated village-level proportions of households who reported they had heard or participated in a given activity. We calculated the means of the village proportions for intervention and control groups and compared them using the Student’ t-test. Within intervention villages, we explored associations between village-level percent awareness of or participation in mobilisation activities and village-level coverage using linear regression. The study was approved by the Ethics Committee at the London School of Hygiene and Tropical Medicine and the Independent Ethics Committee at the Xavier Institute of Management, Bhubaneswar, India. Informed written consent was obtained from the male and/or female head of household prior to the enrolment in the study [6].

## Results

### Context

#### Study setting

The study is a cluster-randomised trial conducted in 100 villages in seven blocks of Puri district in the State of Orissa, India. The study design, population and setting have been described elsewhere [6]. In brief, Puri is located on the coastal area of Orissa and study villages are within easy reach of the capital city of Bhubaneswar. Most people rely on agriculture as the main source of income and 50% own a BPL card. In 2008, sanitation coverage in Orissa was estimated at 15%, while in the district of Puri itself at 23% (DLHS [11]). In Orissa, 67% of rural households rely on tubewells for drinking water.

#### Government of India’s Total Sanitation Campaign

The government of India’s Total Sanitation Campaign (TSC) was initiated in 1999. The programme is implemented at State level under the Rural Development Department. The key components of the programme are: 1) construction and use of individual household latrines, 2) construction of latrines in rural schools, kindergarten and public institutions, 3) provision of low subsidies or ‘incentives’ towards latrine construction to households falling below poverty line (BPL), 4) creation of production centers to provide locally appropriate technologies, and 5) Information, education and communication (IEC) activities designed to generate demand for toilets and encourage use [7]. In 2003, the government of India launched the Nirmal Gram Purashkar (NGP) initiative to stimulate the campaign by providing financial rewards to Gram Panchayats, block and districts who are ‘open defecation free’. In 2012, the TSC was expanded and renamed as Nirmal Bharat Abhiyan. Under the new scheme, the subsidy amount was increased and was provided not only to BPL households, but also to households above the poverty line (APL) who qualify as ‘poor’ [12]. Under the programme’s guidelines, NGOs play a key role by conducting IEC activities and capacity building at the community level and by facilitating hardware supply by operating production centres and rural sanitary marts.

### Intervention

#### Organisation

At the village level, the intervention was delivered by WaterAid and a local NGO partner, United Artist Association (UAA). Six local NGOs were contracted to implement the intervention in seven blocks of Puri district in collaboration with local government. WaterAid was responsible for project oversight, technical support on the project implementation and monitoring. WaterAid also provided funding towards latrine construction for poor households who were not eligible for government subsidy. UAA coordinated implementation activities between the six NGOs and with the local government representatives and relevant departments. Implementing NGOs were assigned between four and twelve villages each. NGOs were selected based their experience with similar community-based projects in the selected areas.

Each NGO appointed one cluster coordinator and village motivators on the basis of one motivator being responsible for two villages. Cluster coordinators were responsible for overseeing implementation of the programme in all assigned villages. Village motivators were recruited from the project area to facilitate mobilisation activities and coordinate latrine construction logistics in villages. Cluster coordinators were typically employees of the NGO while village motivators were often recruited specifically for the project for the duration of one year. Village motivators did not necessarily have extensive experience in community mobilisation or in water, hygiene and sanitation projects. They reported progresses to cluster coordinators on a weekly basis and provided monthly reports.

In February 2011, training sessions were held for village-level implementers. A total of 25 village motivators and 6 cluster coordinators appointed by the NGOs attended a 3-day residential training course organised by UAA. The training covered the key elements of the Total Sanitation Campaign, an introduction to Participatory Rural Appraisal (PRA) concept and tools, communication techniques, technical aspects of latrine construction, roles and responsibilities, and work plan. The training consisted of classroom presentations and group discussions with a half-day field practice on Participatory Rural Appraisal (PRA) and a visit to the production centres. Each NGO selected two ‘Master’ masons who would be responsible for latrine construction and supervision and training of local masons in their allocated villages. Masons received a five-day training course on latrine construction.

#### Hardware

The latrine design consisted of a pour-flush latrine with a single pit and a Y-joint for diversion to a future second pit. At the start of the programme, the contribution of the programme towards latrine construction was set at INR2200 (then approximately US$33). This amount covered the costs of three pit liner rings and cover plate, two bags of cement, one Y-connector, one connector pipe, one ceramic pan set, and one door. This amount also included the cost for transporting the material to the village and 1.5 days of mason’s time. Sand, bricks, stones and two days of labour were to be covered by the household. Village motivators maintained a register containing the material and corresponding costs contributed by each household along with the head of household signature. The value of the contribution made by each household varied but was mostly equivalent to the subsidy amount of INR2200. Construction material such as pipe, pan set, Y-connector, cement were purchased from external suppliers and stored at a central production centres set up at one of the implementing NGOs. The doors were made at the central production centre while the rings and cover plates were produced at ‘satellite centres’ located nearer or within the intervention villages.

#### Community mobilisation

Details of the key components of community mobilisation along with the time frame for each activity are provided in Table 1. In brief, the approach consisted of initial meetings with community leaders to explain the programme, a baseline assessment of the water, hygiene and sanitation and socio-economic profile of the village, the formation of Village Water and Sanitation Committee (VWSC), and a combination of community-level events and door-to-door household visits to encourage construction and use of toilets. Additional IEC activities included wall paintings, school rallies and the formation of adolescent girls groups to disseminate sanitation messages among families and neighbours.

**Table 1:** Key components of the community mobilisation process and timing of activities

**Component**	**Description**	**Dates**
Introductory meetings	NGO cluster coordinator and village motivator meet with local government representatives, key opinion leaders and members of existing community-based organisations such as Self-Help Groups to explain details about the programme.	Feb-Apr 2011
Baseline survey	A second or third meeting is organised the following week to meet with key leaders and provide further details on the programme and collect preliminary information on the village structure, socio-economic profile and water, hygiene and sanitation conditions. During this visit, the village motivator may visit households door-to-door to prepare a list of households with details on BPL status to estimate number of beneficiaries per village. Whenever possible, the BPL list is verified against the BPL list maintain at the Gram Panchayat office.	Feb-Apr 2011
Village Water and Sanitation Committee (VWSC)	The committee is typically composed of 10–15 members. The VWSC includes local government representatives, schoolteacher, kindergarten (*Anganwadi*) worker, community health worker (*Accredited Social Health Activist, ASHA*), villager elders, Self-help group members. At least a third of committee members should be women and lower socio-economic groups and schedule castes should be represented.	Feb-Apr 2011
	The role of the VWSC is to inform community members about the programme and encourage participation, develop an action plan for their village, help with the identification of beneficiaries, liaise with NGO staff and community members to resolve any potential conflicts and issues, support latrine construction logistics such as material procurement and storage, and record keeping.	
	VWSC members attend a 2-day training organised by the implementing NGO and meet once a month thereafter to review progresses with the village motivator and local masons and to discuss and resolve issues arising during the implementation.	
Participatory Rural Appraisal	Transect walk: The village motivator gathers community members in a public place in the village and walk through the village with community members to identify and discuss sanitation related issues, visit open defecation sites, village water sources etc.	Feb – Apr 2011
	Village mapping exercise: The village motivator stimulate discussion about sanitation issues by encouraging community members to draw a map of the village on the ground and use stones, leaves and colour powder to show village landmarks, houses with and without latrines, defecation sites, and water sources.	
	Wealth ranking exercise: village motivator organises a meeting with community leaders and VWSC members at a central location in the village and encourage discussions to help them identify poorest households in their village.	
Door-to-door household visits	Village motivators visit households door-to-door on a weekly basis to explain the programme, encourage participation, and follow-up on latrine construction progresses.	Feb 2011 – Mar 2012
Wall paintings	Wall paintings are located at the entrance of the village or visible location. Paintings typically include the F-diagram showing the transmission pathways for diarrhoea pathogens, breakdown of latrine construction costs and NGO contact details for transparency, and the map of the village as drawn during mapping exercise. One painting planned in each village.	Jan -Mar 2012
School rally	School-aged children are assembled at the village school and walk through the village with placards while chanting slogans about sanitation. One school rally planned to take place in each village.	Jan-Mar 2012
Adolescent girls group or *‘Kumari committee’*	Adolescent girls groups engaged in communicating about good sanitation practices among family and friends, organise village cleaning campaigns. Group members attend a 2-day training organised by the NGO.	Mar 2012

### Fidelity assessment

#### Latrine construction

**Figure 1:**
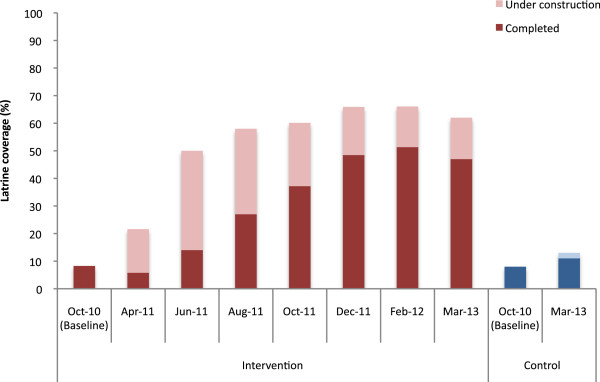
Latrine construction progress from baseline and over intervention period among intervention villages and control villages (n = 4699 households in March 2012 and n = 4585 in March 2013).

**Figure 2:**
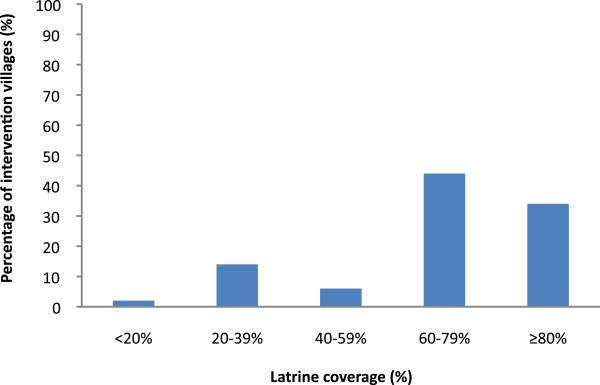
Village-level coverage one year after the start of implementation (March 2012) among intervention villages (n = 50) by quintiles.

At baseline (October 2010), sanitation coverage was similar among intervention and control villages with 8% of households reporting having access to a toilet facility (Figure 1). In March 2012, a year after implementation, 66% of households in intervention villages were observed to have a completed latrine or one under construction. Coverage was higher among BPL than APL households (68% versus 61%, respectively) and ranged from 54% to 76% across the different NGOs. While 39 (78%) intervention villages achieved over 60% coverage one year after the start of implementation, 8 villages (16%) lagged behind with coverage of latrine completed and under construction remaining at less than 40% (Figure 2).

#### Community mobilisation

Information on VWSC formation and composition was obtained for 48 villages. Information was missing for two villages where NGOs encountered delays in implementation due to political issues within the communities. In most villages, committees were established after one or two meetings between February and June 2011. The mean number of members in each committee was 12 (range 5 to 16) and 40% of VWSC members were women. Committees included local government representatives (11%), Self-help group members (16%), kindergarten or community health workers (13%), and teachers (2%). The remaining were key opinion leaders or community members who volunteers to be part of the committee.

Two VWSC members per village were invited to participate in a 2-day training at the NGO office. Each training course had approximately 20–25 participants. The key objectives of the training were to 1) discuss the problems associated with lack of sanitation, 2) explain the objectives of the TSC programme including discussions on latrine construction logistics and contribution costs to ensure transparency, and 3) help committee members to prepare an action plan for their village. The NGO used pile sorting exercises with colour cards to display different behaviours and asks the audience to categorise the behaviours as good or bad and to explain the reasons why. This was followed by a discussion on existing defecation practices in the village and by learning a song on sanitation. The second day covered roles and responsibilities and development of an action plan.

In 37 villages, mapping exercise activities reportedly took place. In six villages, the village motivator reported that no community-level participatory mapping exercise was conducted and information could not be obtained for seven villages. Important differences were noted in the way village motivators described how the mapping exercise was done. In half of the 37 villages, village motivators would describe the mapping exercise as a participatory process where they called on people to a central location in the village and engaged villagers in discussions to draw a map on the floor using colour powder and point out key landmarks in the village, houses, open defecation fields, households with latrines and water sources. The number of participants reported to attend ranged between 15 and 20, and most were VWSC members. In two villages, the motivator reported that 30–45 people attended the event although most people left within one hour. The mapping exercise was typically completed within a half-day including waiting time to gather community members. In three villages, the mapping exercise was reported to have taken 3 days. None of the village motivators mentioned using tools such as faeces counts or standing in open defecation areas as are used in community-led total sanitation (CLTS) programmes. In the remaining villages, village motivators reported that the mapping exercise was conducted with the help of two to three VWSC members and consisted of walking around the village and simply sketching a map of the village on a piece of paper.

Village motivators reported weekly door-to-door household visits. They explained the advantages of having a latrine, provided details of the programme including contribution amounts and construction logistics. They used behaviour change messages provided to them during their initial training. The communication strategy did not focus on a well-defined set of key messages. Instead, sanitation messages were varied and included themes such as inconvenience (at night, time wasted to walk to open defecation sites), women safety and privacy, shame, health, loss of school and work days from being sick, cost of treatment for intestinal infections. Some village motivators carried with them a picture of the latrine design but were not provided with any other communication tools to engage householders in discussions during visits.

According to NGO staff, wealth ranking exercises consisted of organising a meeting with VWSC members and asking them to identify and make a list of households in the village that were considered as poor but did not owned a BPL card. Provision of financial assistance to some but not all households was a frequent source of tensions between the NGOs and communities. As a result, implementers decided to provide a subsidy to all households in intervention villages to prevent delays in the implementation.

As of the last process documentation visit in March 2012, school rallies were recorded to have taken place in 31 villages. School rallies were conducted once during the first quarter of 2012 among children in primary school and included approximately 25–35 students. Village motivators provided teachers with slogans and songs about sanitation and prizes for students who successfully recited them. Children were then given placards and marched through the village while chanting slogans on the merits of sanitation. Wall paintings were observed in 28 villages, although this number is likely to be an underestimation because paintings were being produced during the time of the last visit. Wall paintings typically showed the F-diagram representing the transmission pathways for faecal pathogens. The NGO also included the cost breakdown for latrine construction in order to make the process transparent to the community.

Adolescent girls groups or ‘*Kumari committees*’ were reported to be formed in 31 villages. In 6 villages, no groups were formed as of the last visit and no information was available for the remaining 13 villages. A training course was organised by the implementing NGOs. The content of the course or the actual role of those committees as described by village motivators was vague. Some mentioned that the groups would become engaged in micro-finance activities while others mentioned that the role of the committee was to discourage open defecation, engage in village cleaning activities, and to raise awareness about the issue of sanitation among their family members and neighbours. Village motivators were unclear about the structure of those committees, what they were actually supposed to do and how.

### Exposure to intervention

#### Levels of awareness among community members

Overall, the percentage of households who had heard about the total sanitation campaign was significantly higher in intervention than in control villages (91% versus 49%, respectively, p < 0.001). Perceived benefits associated with having a latrine were broadly similar across intervention and control villages (Table 2). In intervention villages, households heard about the campaign mostly from NGOs (64%) or VWSC members (17%) while in control villages, respondents heard about it from neighbours (30%), NGOs (20%), ward member (15%) or family (12%) and friends (10%). Almost none of the households in intervention villages recalled any form of participatory activities such as transect walk and mapping exercise (6%) or wealth ranking (0%). The proportions were similar in the control villages. However, intervention households were more aware of VWSCs than controls (51% versus 9%, p < 0.001). Awareness of *Kumari committees* was higher among intervention villages (23% versus 8%, p < 0.01). Overall, 36% and 43% of intervention and control households remembered school rallies being conducted in their village. Wall paintings and household visits regarding sanitation over the past three months were also more commonly cited among intervention households (44% versus 7%, p < 0.001 and 65% versus 3%, p < 0.001, respectively). Among the topic being discussed during home visits, intervention households remembered contribution amounts (70%) and latrine construction logistics (52%) the most. Much less remembered discussions around use (26%) and benefits of latrines (20%).

**Table 2:** Awareness of mobilisation activities among intervention and control households (n = 807)

	**Intervention (n = 408)**		**Control (n = 399)**		**Difference**	**p-value**
	**N**	**%**	**N**	**%**	**%**	
Mean age of respondent (sd)	45 (15)		41 (14)			
Respondent is female	249	61	222	56	5	
**Perceived benefits of having a toilet**						
Convenient when it rains or during floods	187	46	193	48	−2	0.61
Time saving from walking to OD sites	241	59	189	47	12	0.02
Health benefits	141	35	114	29	6	0.18
Safety	128	31	131	33	−2	0.82
Prevent contaminating the environment	70	17	87	22	−5	0.15
Convenient at night	118	29	138	35	−6	0.47
Convenient for elderly	46	11	48	12	−1	0.73
Convenient for children	47	12	75	19	−7	0.01
Convenient when sick	19	5	55	14	−9	0.02
Convenient for disabled person	4	1	6	2	−1	0.51
Safer for women	71	17	92	23	−6	0.04
Give privacy to women	82	20	84	21	−1	0.98
Cost saving	2	0	15	4	−4	<0.01
Status improved	8	2	16	4	−2	0.04
Shame	16	4	0	0	4	<0.01
Good for married women	17	4	1	0	4	<0.01
**Heard about sanitation campaign**	373	91	194	49	42	<0.001
**Heard about campaign from (n = 567)**						
NGO	238	64	38	20	44	<0.001
VWSC	63	17	0	0	17	<0.001
Ward member	21	6	30	15	−9	<0.01
Anganwadi worker	12	3	16	8	−5	0.09
ASHA	23	6	0	0	6	0.02
School teacher	3	1	0	0	1	0.09
Adolescent girls committee	3	1	0	0	1	0.19
Self-help group	5	1	9	5	−4	0.06
Neighbours	34	9	59	30	−21	<0.001
Family	10	3	23	12	−9	<0.01
Friends	1	0	19	10	−10	<0.001
**Heard or seen village walk or mapping exercise**	26	6	38	10	−4	0.04
**Heard of wealth ranking exercise**	1	0	5	1	−1	0.09
**Heard of village water and sanitation committee**	207	51	37	9	42	<0.001
Can cite name of at least one VWSC member	169	41	26	7	34	<0.001
Can explain what VWSC members do	138	34	8	2	32	<0.001
**Heard of adolescent girls group**	93	23	33	8	15	<0.01
**Heard or seen school children rally**	147	36	173	43	−7	0.10
**Seen wall paintings**	178	44	28	7	37	<0.001
**Remember content of wall painting (n = 206)**						
Transmission of diarrhoea	103	57	6	21	36	<0.01
Latrine cost breakdown	104	57	2	8	49	0.01
Village map	68	38	3	11	27	0.24
**Received home visit about sanitation in past 3 mo**	242	65	12	3	230	<0.001
**Person who came at last visit**						
NGO staff	257	63	11	3	60	<0.001
VWSC member	13	3	0	0	3	0.21
Ward member	4	1	7	2	−1	<0.01
Anganwadi worker	4	1	1	0	1	0.46
ASHA	12	3	0	0	3	0.36
SHG member	25	6	2	1	5	0.97
**Remember being discussed during last visit**						
Contribution amount	285	70	4	1	69	0.001
Latrine construction logistics	211	52	10	3	49	0.04
How to use and maintain latrine	108	26	2	1	25	0.91
Benefits of having a latrine	80	20	1	0	20	0.88
Inform about meetings	37	9	0	0	9	0.66
Kumari committee	2	0	0	0	0	0.12

*p-values calculated using the t-test on village-level percent awareness of or participation in intervention activities.

#### Awareness among VWSC members

Overall, 57% of VWSC members reported that they were invited to participate in a training course provided by the NGO and 69% of those reported attending the training (Table 3). The topic most remembered was about the benefits of using the latrine (66%) followed by sessions on communication techniques to motivate other villagers to build a latrine (47%). 54% of VWSC members saw their role as encouraging people to construct toilets, but only 21% described being involved in overseeing latrine construction logistics. Even fewer (8%) mentioned their role was about encouraging toilet use. Almost a third didn’t know what their role as VWSC members was. VWSC meetings almost always took place in the presence of the village motivator (89%). Almost half (45%) reported not attending the last VWSC meeting and 40% never conducted door-to-door household visits in relation with the programme.

**Table 3:** Awareness of mobilisation activities among members of village water and sanitation committee of intervention villages (n = 170)

	**n**	**%**
Respondent is female	91	53
Mean age of respondent (SD)	44 (12)	
**Invited to participate in training**	97	57
**Attended the training**	67	69
**Topics remember being discussed at training**		
Benefits of having a latrine	44	66
How to motivate people to build a latrine	30	47
Latrine cost and contribution amounts	21	31
How to motivate people to use latrine	18	27
Instructions on how to construct latrine	11	16
**Perceived role as VWSC member**		
Encourage households to construct latrines	90	54
Oversee latrine construction work	36	21
Encourage households to use latrines	14	8
Conduct meetings	11	7
Don't know	50	30
**Who organises VWSC meetings**		
VM	141	89
Other VWSC members	17	9
**Number of VWSC meetings remembered being held**		
0-4	79	46
5-9	56	33
10+	29	17
**Attended the last VWSC meeting**	94	55
**How often is the village motivator present at those meetings**		
Always	150	93
Sometimes	5	3
Rarely	1	1
Never	5	3
**Ever conducted household visits**	102	60
**Frequency of visits**		
1-4	39	38
5-9	18	17
10+	41	40
**Remember discussing during those visits**		
Instruction on how to construct latrine	86	51
Latrine cost breakdown and contribution amounts	76	45
Benefits of having a latrine	65	39
How to use and maintain a latrine	30	17

We explored if there was any association between awareness of or participation in different mobilisation activities and latrine coverage among households and members of the village water and sanitation committee in intervention villages. There were some evidence that latrine coverage was higher among villages where a larger proportion of households remembered seeing wall paintings (p = 0.05), reported a home visit by the village motivator during the past month (p = 0.02), and among villages where village water and sanitation committee members reported that five or more VWSC meetings were held since the start of the programme (p = 0.04) (Table 4). There was no apparent association between reported awareness of or participation in other activities and latrine coverage.

**Table 4:** Association between village-level coverage in March 2012 and awareness of or participation in mobilisation activities in the 50 intervention villages

	Regression coefficient*	**95%CI**	p-value*
**Household awareness (n = 408)**			
Heard about sanitation campaign	0.203	(−0.306; 0.712)	0.43
Heard or participated in transect walk/mapping exercise	0.637	(−0.104; 1.379)	0.09
Heard or participated in wealth ranking exercise	1.530	(−2.261; 5.321)	0.42
Heard of village water and sanitation committee	0.181	(−0.660; 0.428)	0.15
Heard of adolescent girls groups or kumari committees	0.233	(−0.051; 0.518)	0.11
Heard or seen school children rally	0.230	(−0.025; 0.482)	0.07
Seen wall paintings	0.171	(0.001; 0.341)	0.05
Village motivator visited their house in the past month	0.216	(−0.000; 0.431)	0.05
**VWSC members awareness (n = 170)**			
VWSC members attended NGO training	0.001	(−0.181; 0.183)	0.99
≥ 5 VWSC meetings held since the start of the programme	0.178	(0.010; 0.346)	0.04
VWSC attended the last VWSC meeting	0.060	(−0.164; 0.284)	0.59
VWSC member ever conducted household visits	0.025	(−0.205; 0.254)	0.83
VWSC member conducted ≥ 5 household visits	0.058	(−0.156; 0.272)	0.59

*Regression coefficients express increase in latrine coverage in percent with every additional percent increase in awareness of or participation in activities among respondents in a village and among VWSC members.

## Discussion

Our process documentation of a large rural sanitation programme under the umbrella of TSC revealed differences between what was planned according to TSC guidelines and what was actually done on the ground. Targets set by implementing and supervising NGOs were by and large, not achieved.

Between January 2011 and January 2012, coverage of completed latrines among the fifty intervention villages increased from 8% to 51%. This is lower compared to the initial target of 90% coverage set by the implementing NGOs at the outset of the project. The implementing organizations reported a number of challenges that impacted on the delivery of the programme. First, in June 2011, the subsidy amount was increased from Rs2200 to Rs3200. As a result, some communities who heard about the raise complained to the NGOs that they were entitled to more money towards construction. Second, a road construction project was taking in Puri district at the same time of implementation. Householders, who had to contribute sand towards latrine building, complained about the increase in the price of sand due to competition with the road project and decrease in availability. Third, several masons initially trained at the start of the programmes left for better paid construction work elsewhere, which disrupted construction and impaired the quality of construction. Fourth, heavy rain and floods caused delays in construction. In September 2011, 13 villages were severely affected by floods and could not be accessed for several weeks. Fifth, activities had to be interrupted for two months as part of the code of conduct governing elections and political unrest in four of the intervention villages prevented any construction activities whatsoever.

Despite the shortfall from the target coverage level, the actual level of coverage achieved was comparable to those observed elsewhere under the TSC, suggesting that the roll out of the intervention may have been by no means untypical of current sanitation activities in rural India. According to the latest figures, sanitation coverage in Orissa increased from 8% in 2001 to 59% in 2011–2012 [12]. Another study in a coastal district of Orissa reported a rise from 6% to 32% among 20 intervention villages within a one year period [13]. In Tamil Nadu, coverage increased from 15% to 48% five years after the start of the programme [14]. However, in the present study, we found differences between villages. While 26 villages achieved latrine coverage levels above 60%, 13 villages achieved less than 40% a year after the start of implementation. One intervention village remained essentially at baseline coverage because of local resistance to implementation; others made only modest gains in coverage due to reasons such as lack of interest and cooperation from the village water and sanitation committee members or key community members, village motivators leaving the programme resulting in villages being visited less frequently; and disputes between NGO staff and community leaders regarding construction logistics, material provision and masons payments. Once again, this variability is characteristic of TSC implementation generally [15]. It is possible that latrine coverage can increase over time after the implementation of the TSC. A cross-sectional survey among twenty villages in the same district where WaterAid implemented TSC three years ago reported a latrine coverage of 72% [16].

While the TSC targets BPL households, we found that the levels of latrine coverage achieved were only modestly (7%) higher among BPLs than APLs. This may be due to the fact that as implemented in these villages, TSC subsidies were provided to all households instead of BPL card owners and ‘poor’ households only. However, this is not entirely inconsistent with the TSC in States such as Bihar and Chhattisgarh that have made provisions for subsidies for APL households from their own funds [15].

One year after implementation, the overall level of awareness of TSC was higher among intervention than controls villages. However, almost half of interviewed households in control villages had already heard about the programme. While two thirds of respondents in intervention villages said they heard about it through NGOs, the majority in control villages reported they heard about it through family, friends, neighbours, or ward or PRI members. It is possible that information about the programme spread from intervention to control villages. This may also be due to study villages being located in a district close to the State capital city where people may already have more knowledge about existing government programmes.

Although implementers reported that the intervention was designed to include a participatory ‘triggering’ approach, interviews of village motivators revealed that those activities were either not conducted or conducted with a few individuals and little community involvement. This explains why almost none of the intervention households recalled hearing of or participating in activities such as transect walk or mapping exercise. However, formation of a VWSC and frequent home visits were frequently mentioned. Half of interviewed households knew about the existence of a VWSC and two third reported a home visit in the past three months, mostly by the village motivator, to discuss matters related to sanitation. However, a vast majority remembers contribution amounts and construction logistics as the main topics being discussed. Although a village water and sanitation committee was formed in each village, its members did not attend to meetings regularly and implementation often depended on a few dedicated committee members. Similar problems with village water and sanitation committees have previously been highlighted [17].

Some of the key perceived benefits associated with having a toilet included convenience when it rains or during flood, time saved from walking to open defecation sites, health benefits, safety and privacy for women. Health as well as safety and privacy for women have been also cited as key advantages in similar contexts [16,18]. Overall, perceived benefits were broadly similar between treatment arms. The communication messages designed to encourage households to build and use toilets consisted of more than ten different themes ranging from health to convenience or avoiding shame. However, studies have suggested that health is often not a key driver for hygiene behaviour change and that using fewer key messages may be more effective [19,20]. Avoiding shame was almost never mentioned as a benefit of having toilets, suggesting failure of the intervention to impact on social norms that do not perceive open defecation as shameful.

There were a number of challenges associated with collecting data on the implementation process. Our process documentation team was separate from the implementing organisations in an attempt to preserve some degree of independence from the implementers. Unlike other process evaluations designed to optimise implementation as it is being rolled out, we wanted to minimise our influence in order to measure health impact in a programmatic setting. This sometimes resulted in tensions between the monitoring teams and implementers who felt were being scrutinised. For example, obtaining exact figures on the number of households in each village was not always easy and resolving discrepancies between implementing and monitoring teams’ records was challenging at times. We defined a household as a group of individuals living under the same roof and sharing the same cooking pot (NFHS, [21]). However, households in our study area are mostly joined families and the definition was difficult to apply. On several occasions, enumerators and implementation teams complained that households over or under-reported number of families living under the same roof depending on which team visited them and whether they thought they could receive financial benefits or had to contribute towards costs of latrine construction.

The process documentation team could monitor outcomes such as latrine coverage independently from the implementers, but had to work closely with village motivators and NGOs to collect information on mobilisation activities. Village motivators maintained a log of their work and were encouraged to do so rigorously by the NGO. However, the completeness of the logbook varied largely from one village motivator to another. Therefore, information was sometimes missing or it was impossible to verify information against written records. This was especially problematic when village motivators left the programme and were replaced.

## Conclusion

In conclusion, our process documentation suggests that implementation of TSC in our study area falls short of the TSC guidelines. The levels of coverage achieved and the levels of awareness of the mobilisation process in our intervention villages were far lower than planned, but similar to those reported elsewhere in India under the TSC. This suggests that the findings from our trial may apply to other settings in India where TSC has been rolled out. Whether the NBA programme (TSCs successor) will achieve better results remains to be seen. Our process evaluation study suggests that subsidies alone are unlikely to solve India’s sanitation problem as long as village mobilisation remains patchy, and the motivations and social norms of open defecation remain unaddressed.

## Competing interest

The authors declared that they have no competing interest.

## Authors’ contributions

SB, PS, TC designed the protocol for process evaluation. WS and SR contributed to the data management and analysis. PR, SB and BT implemented data collection. All authors read and approved the final manuscript.
